# Cardiac Complications in Systemic Lupus Erythematosus: A Systematic Review of Diagnostic and Prognostic Gaps

**DOI:** 10.7759/cureus.102966

**Published:** 2026-02-04

**Authors:** Raghavee Neupane, Brahadesh Sivakumar, Ebin Mathew, Marc M Kesselman

**Affiliations:** 1 Medicine, Nova Southeastern University Dr. Kiran C. Patel College of Osteopathic Medicine, Fort Lauderdale, USA; 2 Rheumatology, Nova Southeastern University Dr. Kiran C. Patel College of Osteopathic Medicine, Davie, USA; 3 Rheumatology, Nova Southeastern University Dr. Kiran C. Patel College of Osteopathic Medicine, Fort Lauderdale, USA

**Keywords:** biomarkers, cardiac mri, cardiovascular disease, lupus flare, myocarditis, pericarditis, risk stratification, subclinical cardiac involvement, systemic lupus erythematosus (sle), underdiagnosis

## Abstract

Systemic lupus erythematosus (SLE) is a chronic autoimmune disease with multisystem involvement and fluctuating activity. Among its many complications, cardiovascular disease (CVD) is a leading cause of morbidity and mortality, with up to 25% of patients affected. Cardiac manifestations, such as pericarditis, myocarditis, and accelerated atherosclerosis, are often underdiagnosed due to their subclinical presentation and lack of standardized screening protocols. This systematic review aims to synthesize current evidence on the incidence, detection, and prognostic challenges of cardiac complications in SLE patients and to identify gaps in current risk stratification and diagnostic methods. A comprehensive literature search was conducted across Ovid, CINAHL, EMBASE, and Web of Science using predefined search terms related to SLE, cardiovascular complications, disease flares, and diagnostic tools (e.g., biomarkers, cardiac MRI). English-language studies published between 2015 and 2025 involving adults (≥18 years) were included. Eligible studies focused on cardiac complications in SLE patients and reported outcomes such as incidence, diagnostic markers, or evidence of underdiagnosis. Exclusion criteria included pediatric populations, non-SLE autoimmune diseases, lack of cardiac outcome data, animal/in vitro studies, non-English papers, reviews, and editorials. Across 31 studies encompassing over 7,000 patients with SLE, cardiac symptoms were frequent, ranging from subclinical abnormalities to life-threatening complications. Case reports highlighted severe manifestations, including cardiac tamponade, myocarditis, cardiogenic shock, and myocardial infarction, often mimicking acute coronary syndromes. Observational and retrospective cohorts demonstrated high rates of pericardial effusion (up to 25%), pulmonary hypertension (15-42%), valvular disease (15-32%), and subclinical myocardial dysfunction detected by advanced echocardiographic or PET modalities. Large-scale cohorts identified lupus myocarditis in 1.7%-9% of patients, strongly associated with higher Systemic Lupus Erythematosus Disease Activity Index (SLEDAI) scores, hypocomplementemia, and anti-nucleosome antibody positivity. Biomarker studies reported elevated troponin, IL-18, IL-1Ra, and interferon-α as predictors of myocardial injury and adverse outcomes. Mortality data linked poor prognosis to myocarditis, pulmonary hypertension, renal disease, and higher comorbidity indices. Outcomes varied, and patients were treated with diverse therapies, including glucocorticoids, immunosuppressants, biologics, and cardiovascular medications. Collectively, findings underscore that cardiac manifestations in SLE are common, multifactorial, and frequently underdiagnosed, with biomarkers and advanced imaging emerging as valuable tools for early detection. Cardiac complications in SLE are prevalent, diverse, and often underrecognized. This review highlights the critical need for improved diagnostic strategies, including the use of emerging biomarkers and advanced imaging techniques, to enable earlier detection and more accurate risk stratification. Standardizing screening protocols and incorporating cardiovascular assessment into routine SLE management may reduce delays in diagnosis and improve patient outcomes. Further research is essential to develop consensus guidelines for early identification and prognostication of cardiac involvement in SLE.

## Introduction and background

Systemic lupus erythematosus (SLE) is a chronic autoimmune disease characterized by multisystem involvement and fluctuating disease activity. SLE commonly affects women 10 times more than men, especially middle-aged women of childbearing age [1]. Its prevalence is disproportionately greater in African American, Asian, and Hispanic populations [1]. Additionally, SLE is correlated with cigarette smoking, oral contraceptive use, and hormone replacement therapy for post menopause [2]. SLE is known to lower the quality of life for patients to a similar level as patients with hypertension, arthritis, osteoporosis, hyperlipidemia, and much more [3]. The disease is characterized by the deposition of antigen-antibody complexes, which cause a wide range of tissue damage. Common complications from SLE include psychiatric, vascular, lymphatic, cardiac, and renal issues.

Among its many manifestations, cardiovascular complications represent a major source of morbidity and mortality, as 25% of SLE patients suffer cardiac complications [4]. This statistic represents the overall reported prevalence of cardiac complications in SLE populations. According to the National Institutes of Health (NIH), cardiovascular disease (CVD) accounts for 33% of SLE-related deaths and is 3.63 times more likely to be the underlying cause of death in patients with SLE compared to the general population [5]. This statistic reflects the proportion of SLE-related deaths attributable to CVD, rather than the point prevalence of subclinical or clinically overt cardiac pathology. Cardiac manifestations in SLE include pericarditis, myocarditis, and Libman-Sacks endocarditis (a form of sterile valvular inflammation), accelerated atherosclerosis, arrhythmias, and heart failure. They are frequently underdiagnosed due to their subclinical nature and the absence of standardized screening strategies [4]. These complications often appear at an earlier age due to accelerated atherosclerosis and lead to greater levels of chronic inflammation [6].

The primary concern is that cardiac complications can be subclinical and remain undetected until advanced stages. Diagnostic limitations, such as the absence of standardized screening protocols, further contribute to delayed recognition [7]. Echocardiography presents as an impediment because it is difficult to reproduce, tissue characterization cannot be completed on SLE patients, and the quality of images through acoustic windows may be impaired [7]. Even common methods, such as a coronary angiogram for diagnosing CVD, are ineffective; studies have shown that a coronary angiogram was unable to show inadequate blood flow in up to two-thirds of SLE patients presenting with it [6].

Despite the growing recognition of cardiac symptoms in SLE, significant diagnostic and prognostic uncertainties persist. Currently, there is no consensus on the optimal risk stratification, early detection, or prediction of disease progression in patients affected by this condition. One limitation of current risk stratification is the lack of a singular threshold for defining low, moderate, or high risk. Higher non-calcified plaque volume has been linked to increased plaque vulnerability and heightened vascular inflammation [8]. Potential future manifestations from uncalcified plaque are not captured by coronary artery calcification (CAC) assessment, thereby indicating the need for a synthesized diagnostic and prognostic criterion [9]. This systematic review aims to synthesize the literature on cardiac complications in SLE, with a specific focus on identifying and characterizing the diagnostic and prognostic gaps that hinder optimal patient care.

The objective of this review is to differentiate between subclinical cardiac conditions and clinically overt cardiac events. Subclinical cardiac involvement is defined as abnormalities detected by imaging or biomarkers in the absence of overt cardiopulmonary symptoms. Clinically overt events are characterized by symptoms such as shortness of breath, chest pain, arrhythmias, heart failure, or presentations consistent with acute coronary syndrome. This differentiation is required because many cohorts found abnormalities predominantly by echocardiography instead of clinically overt symptoms, while case reports and series focused on acute, high-severity presentations, such as myocardial infarction, myocarditis, cardiac tamponade, and cardiogenic shock.

## Review

Methods

A systematic literature review was performed using Ovid, CINAHL, EMBASE, and Web of Science using the search terms ("systemic lupus erythematosus") AND (“disease flare” OR “disease exacerbation”) AND (“acute coronary syndrome” OR “myocardial infarction” OR “silent ischemia”) AND (“incidence” OR “underdiagnosis” OR “biomarker” OR “troponin” OR “heart magnetic resonance imaging” OR “cardiac mri”). To ensure the recency of the articles, only articles in English published between 2015 and 2025 involving human participants were assessed. The articles were analyzed in a step-wise process by evaluating the title and abstract for relevance and then assessing the full-text manuscript. The Nova Southeastern University (NSU) library database was utilized to access databases and full-text articles.

For this review, eligible study designs included randomized controlled trials, cross-sectional studies, observational studies, longitudinal studies, case reports/series, and prospective or retrospective cohort studies. Studies were included if they involved adults (≥18 years) diagnosed with SLE based on established classification criteria, such as positive antinuclear antibody (ANA), anti-double-stranded DNA (anti-dsDNA), or anti-Smith (anti-Sm) antibodies. Studies were eligible if they reported cardiac complications in patients with SLE, regardless of flare status, and provided relevant outcomes such as the incidence or prevalence of cardiac events, cardiac biomarkers, imaging findings, or evidence of underdiagnosis.

Studies were excluded if they focused on pediatric populations, non-SLE autoimmune diseases, or cardiac events not temporally associated with flares. Additional exclusions included studies lacking clear cardiac outcome definitions, reporting only general cardiovascular risk factors, animal or in vitro studies, non-English publications, conference abstracts, editorials, or reviews without extractable original data.

**Figure 1:**
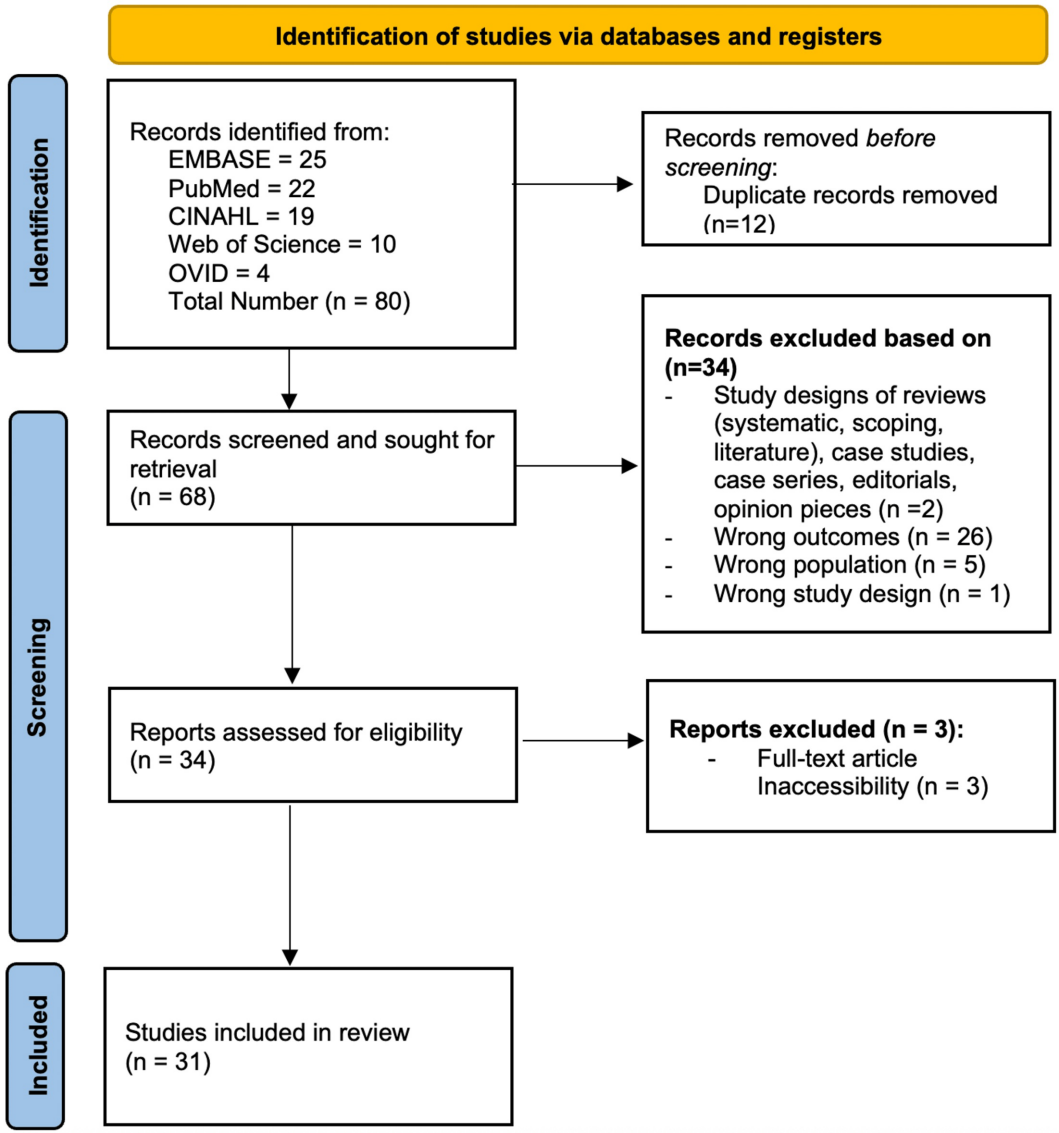
Preferred Reporting Items for Systematic Reviews and Meta-Analyses (PRISMA), indicating data selection

Two reviewers independently screened titles and abstracts, followed by full-text assessment to determine eligibility. Any disagreements were resolved by a third reviewer. Risk of bias was independently assessed by two reviewers using the Joanna Briggs Institute (JBI) critical appraisal tools appropriate to each study design [10]. The Preferred Reporting Items for Systematic Reviews and Meta-Analyses (PRISMA) guidelines were followed to develop a flow diagram of the selection criteria for reproducibility (Figure 1). Due to substantial heterogeneity in study design, patient populations, outcome definitions, and reporting, a meta-analysis was not performed.

Results

Of the included studies, 13 were case reports, one was a case series, six were cross-sectional, three were case-control, and eight were cohort studies (prospective, retrospective, or longitudinal).

Study Characteristics

Case reports highlighted rare or severe cardiac manifestations in patients with SLE, while cross-sectional and retrospective studies provided broader estimates of prevalence. Sample sizes ranged from single-patient case reports to large cohorts of over 700 individuals. The majority of participants were female, aligning with the known sex distribution of SLE. Mean ages ranged from the late 20s to mid-30s, and disease durations ranged from several months to more than five years. Most studies were conducted in diverse international settings, including South Asia, North Africa, and East Asia, emphasizing the global significance of SLE-related cardiac disease. An overview of study characteristics, reported findings, and risk-of-bias assessments for cardiac complications in SLE, conducted using the JBI critical appraisal tools, is provided in Table 1.

**Table 1:** Cardiac complications in SLE: summary of study characteristics, findings, and risk-of-bias assessment using the JBI Critical Appraisal Tools APS: Antiphospholipid Syndrome; AST: Aspartate Aminotransferase; BNP: B-type Natriuretic Peptide; CAC: Coronary Artery Calcification; CAV: Coronary Artery Vasculitis; CCI: Charlson Comorbidity Index; CK: Creatine Kinase; CK-MB: CK–Myocardial Band; CMR: Cardiac Magnetic Resonance Imaging; CMP: Comprehensive Metabolic Panel; CRP: C-reactive Protein; CCTA: Coronary Computed Tomography Angiography; DD: Diastolic Dysfunction; DSV: Diffuse Subendocardial Vasculitis; EDV: End-Diastolic Volume; EDVI: EDV Index; EF: Ejection Fraction; ENA: Extractable Nuclear Antigen; ESImax: Max End-Systolic Strain; ESL: Early Systolic Lengthening; ESR: Erythrocyte Sedimentation Rate; ESV: End-Systolic Volume; ESVI: ESV Index; FDG-PET: Fluorodeoxyglucose Positron Emission Tomography; GLS: Global Longitudinal Strain; HDL: High-Density Lipoprotein; HF: Heart Failure; hs-TropT: High-sensitivity Troponin T; IFN-α: Interferon Alpha; IL-1Ra: Interleukin-1 Receptor Antagonist; IVUS: Intravascular Ultrasound; LA: Left Atrium; LAC: Lupus Anticoagulant; LDH: Lactate Dehydrogenase; LDL: Low-Density Lipoprotein; LM: Lupus Myocarditis; LV: Left Ventricle; LVMI: LV Mass Index; MSSA: Methicillin-Sensitive *Staphylococcus aureus*; NT-proBNP: N-terminal pro–BNP; PAH: Pulmonary Arterial Hypertension; pANCA: Perinuclear Antineutrophil Cytoplasmic Antibody; PGA: Physician Global Assessment; PSImax: Max Peak Systolic Strain; PSS: Postsystolic Shortening; RV: Right Ventricle; SCD: Sickle Cell Disease; SDI: Systemic Lupus International Collaborating Clinics Damage Index; SELENA-SLEDAI: Safety of Estrogens in Lupus Erythematosus National Assessment–SLE Disease Activity Index; SLEDAI: SLE Disease Activity Index; SLEDAI-2K: Systemic Lupus Erythematosus Disease Activity Index–2000; sST2: Soluble Suppression of Tumorigenicity-2; STEMI: ST-Elevation Myocardial Infarction; TNF-α: Tumor Necrosis Factor Alpha; tPA: Tissue Plasminogen Activator; VDRL: Venereal Disease Research Laboratory Test; Vfib: Ventricular Fibrillation

Author (Year)	Study Design	Patients (M/F)	Mean Age	Mean Disease Duration	Cardiac Complication	Diagnostic Modality	Medical History	Medications	Clinical Presentation	Additional Findings	Biomarker(s) Reported	Risk of Bias	Conclusion
Bhatt et al. (2023) [11]	Case Report	1 F	35	Stage 4 lupus nephritis	Cardiac Tamponade	Chest X-ray, Echocardiogram	Lupus Nephritis, Graves' Disease, HTN, Vasculitis, Anemia	Glucocorticoids (high doses), Nifedipine, lisinopril, atenolol, methimazole, mycophenolate mofetil, hydroxychloroquine, voclosporin, belimumab	Seizures, cough, vomiting, diarrhea	Sputum culture: Hemophilus Influenzae; Complications = Acute renal failure, Lung collapse, Recurrent Pericardial Effusion	Not Mentioned	Low	Highlights the importance of promptly identifying and managing pericardial effusion in SLE patients
Lodha et al. (2025) [12]	Case Report	1 M	28	3 months	Myopericarditis	ECG, Echocardiogram, CMP and X-ray	Microcytic Anemia	Colchicine, Prednisone, Hydroxychloroquine, Oxycodone	Chest pain (7/10), Shortness of Breath, Lower Leg Pain	Diffuse ST Elevations on ECG, Pleuritic Friction Rub	Anti-RNP antibody, ANA antibody, Chromatin antibody, Anti-SSA antibody, elevated troponin levels	Low	SLE myopericarditis can mimic ACS (chest pain, troponin rise, ST-elevation); High risk of missed diagnosis due to overlap with ACS and high baseline coronary disease risk in SLE
Kini et al. (2017) [13]	Cross-sectional	50	27.9	71.7 months	Pulmonary HTN (42%); Valvular abnormalities (32%); Pericardial effusion (18%); Systolic dysfunction (8%); Diastolic dysfunction (12%)	Echocardiogram	58% had ≥1 Atherosclerosis Risk Factor	Immunosuppressives	Dermatological (88%), Renal (22%), Pulmonary (6%), Musculoskeletal (52%), CNS (8%), GIT (6%), Hematological (32%), Thyroid abnormalities (22%)	Not Mentioned	ANA, Anti Ds-DNA	Low	Cardiac abnormalities (pericardial effusion, pulmonary hypertension, diastolic dysfunction) are common and often asymptomatic in SLE. So, echocardiography is recommended in those patients. Steroids may increase atherosclerosis risk; early steroid-sparing therapy is advised.
Mohamed et al. (2019) [14]	Cross-sectional	59 (86.4% F)	31.3±10.5	5.18 ± 4.1 years	Pericarditis (3.4%); Pericardial effusion (13.6%); Pericardial thickening (6.8%); Pulmonary arterial HTN (PAH) (8.5%); LVEF <54% (3.4%)	Echocardiogram	Systolic HTN (5.1%); Diastolic HTN (35.6%)	Methotrexate (13.6%); Hydroxychloroquine (88.1%); Azathioprine (55.9%); Corticosteroid (69.5%); Cyclobenzaprine (27.1%); Statins (5.1%); LDA (50.8%); Anti-HTN (40.7%); Anti-coagulant (20.3%)	Not Mentioned	Median (IQR); SLEDAI scores: 13 (8-20); ESR (1st hour): 34 (20-73) Cholesterol (gm/dl): 153 (131- 216) LDL (gm/dl): 85 (63.5- 116.5) HDL (gm/dl): 51 (37- 65) Triglycerides (gm/dl): 95 (73.5- 150), Serum Uric Acid (gm/dl): 4.1 (3.8-5.2)	Anti-dsDNA, C3 and C4 levels	Low	Identified echocardiographic features and clinical predictors of cardiac pathology in SLE to improve risk stratification and prognosis
Jia et al. (2018) [15]	Retrospective	750 (88.8% F)	36.59 ± 14.52	(4.72 ± 5.94) years	Pericarditis (9.5%), Myocarditis (5.7%), Heart valve disease (15.6%), Arrhythmia (16.67%), and cardiovascular diseases (14%); Pulmonary Arterial HTN (PAH) (15.7%)	Transthoracic Doppler-Echocardiography	Not Mentioned	Not Mentioned	Not Mentioned	Mild SLEDAI protected against Myocarditis, moderate SLEDAI increased Arrhythmia risk, age predicted PAH, while remission protected against PAH	anti-ACA antibody (5.3%), anti-SSA antibody (51.7%), anti-SSB antibody (15.6%), anti-dsDNA antibody (52.5%), anti-Sm antibody (30.5%), ANA (80.4%), troponin	Low	In Han Chinese SLE patients, cardiac symptoms are common and correlate with age and disease activity. PAH and myocarditis are risk factors for mortality in SLE
Elsaygh et al. (2024) [16]	Case Report	1 F	33	Not Mentioned	Pericardial Effusion and Tamponade	EKG, chest X-ray, CT, and Transthoracic Echocardiogram	Sickle cell disease (SCD)	Hydroxychloroquine, Mycophenolate, Steroids, Rituximab	Progressive Chest Pain, Polyarthralgia, and Limb Swelling for three days, Fever, Tachycardia, Systolic Murmur, Bibasilar Rales, Bilateral Shoulder and Hip Tenderness, and Polyarticular Effusions	Not Mentioned	Normal procalcitonin and troponin levels	Low	Pericardial effusion and tamponade during an SLE flare complicates managing of these conditions
Alian et al. (2019) [17]	Retrospective	152 (88.2% F)	37.6+10.8	3.8+2.6 years	Myocardial infarction (2.6%), Congestive Heart Failure (6.6%), Peripheral Vascular Disease (2.6%)	Not Mentioned	Serious infections (27%); HTN (23%); Renal disease (12.5%); Peptic ulcer (9.9%); Diabetes mellitus (9.9%); Dyslipidemia (17.8%); Osteoporosis (22%)	Steroid (oral or parenteral) (94.1%); Cyclophosphamide (42.8%); Mycophenolate mofetil (44.7%); Antimalarial (100%)	Not Mentioned	33.3% of early deaths were due to cardiovascular events. Deceased patients (n = 12) were older at diagnosis with shorter disease duration. Mortality was higher in males. Deceased patients had higher rates of lupus nephritis, HTN, dyslipidemia, serious infections, and tumors (P < 0.05)	ESR: 55.0 ± 23.9; anti-dsDNA: 44.7%; SLEDAI: 7.2±5.0 SDI: 2.5±1.6	Low	Mortality was linked to higher SLEDAI, SLICC/ACR Damage Index (SDI), and Charlson Comorbidity Index (CCI) scores. Hypertension, renal disease, hyperlipidemia, infection, and tumors further increased the risk
Liu et al. (2022) [18]	Retrospective case control	4719 (89% F)	32.38 ± 13.55	Not Mentioned	Lupus myocarditis (1.67%), Abnormal Ventricular Wall Motion Function (66%), Decreased LV Ejection Fraction (LVEF) (<50%) (38%)	Echocardiogram	Not Mentioned	Not Mentioned	Not Mentioned	Lupus myocarditis patients had more neurological involvement, higher SLEDAI-2K scores, and more positive anti-nucleosome antibodies than controls	Low C3 and high SLEDAI-2K were independent risk factors for lupus myocarditis (OR=0.870, p=0.041; OR=1.058, p=0.023)	Low	Myocarditis in SLE is rare but serious; awareness of lupus myocarditis after cardiac manifestations may help reduce mortality.
Kumar et al. (2019) [19]	Retrospective	12 (91.67% F)	25.1 ± 8.2	1 month	LVEF <35%; 25% had Pericardial Effusion	Echocardiography	Hypothyroidism (33%)	Methyl prednisolone pulse (66%); Oral prednisolone (75%); IV Immunoglobulins (25%); Intravenous Cyclophosphamide (16%); Mycophenolate Mofetil (25%)	Tachycardia (100%), Dyspnoea, Fever and Arthralgia (66% each)	Median SLEDAI score was 18.8 ± 11.6	mean serum CRP (55.6 ± 41.5 mg/l) and ESR (50.5 ± 28.6 mm/hr) levels; Troponin I elevated (75%); hypocomplementemia	Low	Consider Lupus myocarditis in SLE with dyspnea/tachycardia; Treat with high-dose steroids
Brenes-Castro et al. (2023) [20]	Case Report	1 F	26	18 years	Inferior ST-elevated myocardial infarction (STEMI) with extension to right precordial leads; Left coronary artery ectasia with "string-of-beads" appearance and a total coronary occlusion of the right coronary artery. Coronary artery vasculitis (CAV)	Transthoracic Echocardiogram	Lupus Nephritis II	Prednisone and Hydroxychloroquine	Acute Onset Typical Angina	Coronary Artery Vasculitis (CAV) Primary Finding: "String-of-beads" pattern on CCTA or angiography. Further Characterization: IVUS, MRI: Detail lesion structure. FDG-PET: Best for assessing inflammation in large-vessel vasculitis.	High titers of anti-dsDNA; high-sensitive troponin T; N-terminal pro B-type natriuretic peptide (NT-proBNP); elevated inflammatory markers (CRP, WBC)	Low	Despite detecting CAV, ACS occurred; early noninvasive imaging in SLE may enable timely intervention, but further research is needed to optimize diagnosis and treatment.
Minderytė et al. (2022) [21]	Case Report	1 M	36	12 years total but went into remission 7 years from diagnosis	Cardiac arrest/ST-elevation MI/Vfib	Electrocardiogram, Echocardiogram, Cardiac MRI	SLE (in remission and was not taking meds 1 year prior to MI)	Steroids, Hydroxychloroquine, Warfarin	Chest Pain	Hospitalization: Diagnosed with dyslipidemia and antiphospholipid syndrome (APS). CRP dropped sharply (159.5 → 11.5 mg/L) on hydroxychloroquine & prednisone. Imaging showed: LV apical damage, reduced contraction, LVEF 34% (MRI), post-ischemic changes, and apical thrombus. 1-Month Follow-Up: Echo showed: Hypokinetic septum, LVEF ~40%, and impaired diastolic function.	Troponin, lipid panel, CRP, antibody panel: anti-dsDNA, anti-B-glycoprotein IgG/IgA/IgM, anti-cardiolipin IgG/IgA/IgM, extractable nuclear antigen (ENA), anti-Sm	Low	Autoimmune inflammation leading to severe cardiac pathology may be present even in SLE remission. Minimal doses of aspirin and steroids may be considered in SLE remission, as the patient had been discontinued prior to MI.
Aldarmaki et al. (2021) [22]	Retrospective Chart Review	91 (96.7% F)	26.6 (age at diagnosis)	10.2 years	Specific complications not mentioned, except ACS/CHF after hospitalization	Not Mentioned	Not Mentioned	Medications at the time of admission: Hydroxychloroquine: 84%, Corticosteroids: 69%, Mycophenolate mofetil: 34%, Azathioprine: 30%. New treatments initiated during admission: Rituximab: 3.6% (most common new treatment), Cyclophosphamide: 1.4%. Additionally, 16% of patients received high-dose pulse steroid therapy (methylprednisolone ≥250 mg).	Not Mentioned	Hospitalization rate: 29.5%. Admissions due to lupus flare: 32%. Average length of stay: 5.9 days. ICU admission rate: 7%	ANA (98%), anti-dsDNA (84%), anti-Sm antibody (14%), anti-phospholipid antibodies (16%), low complements (59%)	Low	Lupus flare was the most prevalent cause for hospitalization in this cohort.
Catoggio et al. (2014) [23]	Retrospective chart review	1480. Onset of SLE <50: 1378 patients (90% F). Onset of SLE ≥ 50: 102 patients (87% F)	Onset of SLE <50: 26.5. Onset of SLE ≥ 50: 56.0	Mean SLEDAI in onset of SLE <50: 7.6, mean SLEDAI in onset of SLE ≥ 50: 6.3	Onset of SLE <50: pericarditis (18%), endocarditis (0.15%), myocarditis (4%). Onset of SLE ≥ 50: pericarditis (18%), myocarditis (9%)	Not Mentioned	Varied	Onset of SLE <50: Antimalarials (82%), Corticosteroids (95%), Cyclophosphamide (38%), Azathioprine (32%), Methotrexate(11%), Intravenous Immunoglobulin (1%), Plasmapheresis (1%). Onset of SLE ≥ 50: Antimalarials (84%), Corticosteroids (87%), Cyclophosphamide (20%), Azathioprine (21%), Methotrexate(16%), intravenous Immunoglobulin (1%)	Varied	Late-onset lupus is associated with higher cardiovascular risk (OR 1.76, 95% CI 1.04–2.98).	Not Mentioned	Low	Late-onset SLE has unique clinical manifestations with milder SLEDAI scores and renal/cutaneous involvement but carriers higher risk of cardiovascular, pulmonary, and ocular involvement compared to earlier onset SLE.
Hoff et al. (2019) [24]	Case report	1 F	30	4 years	LV pseudoaneurysm, heart failure syndrome, pericardial effusion	Electrocardiogram, Chest Radiograph, 2D Transthoracic Echocardiogram, Cardiac MRI	Diagnosed with SLE at 26 but lost to follow-up	Discharged with Prednisone 1 mg/kg and hydroxychloroquine and received three monthly pulses of 1 g of cyclophosphamide, followed by azathioprine as maintenance therapy.	1 month history of Dyspnea on Exertion, Orthopnea, and Pleuritic Chest Pain, Fever (7 days), and mild hemoptysis (1 episode).	Low voltage ECG, basal pulmonary rales, splenomegaly. Enlarged cardiac silhouette on chest radiograph. 2D echo showed LV apex akenesis with rupture connected with pericardial fluid collection. 48% LV EF, Anemia of chronic disease, leukopenia. Histological findings: diffuse fibrosis, vascular proliferation, lymphomononuclear inflammatory infiltrate in connective tissue, organizing thrombi on the inner surface, Obliterative intimal thickening and circumferential lymphocytic inflammatory infiltrate in vessel walls.	hemoglobin, leukocytes, high CRP; platelets level, creatinine, electrolytes, glucose, albumin, coagulation tests; urinalysis. Positive for antinuclear antibodies (1/320 nuclear speckled); VDRL and serologies for HIV and Chagas disease	Low	Highlights LV aneurysm as rare but possible complication in SLE patients with normal coronary arteries. Patient had completed resolution of symptoms after discharge and LV EF rose to 65% after 1 yr follow-up.
Perel-Winkler et al. (2018) [25]	Case Series	6 F, 2 M	43	11 years	Myocarditis, coronary artery disease, pericardial effusion, LA dilation, NSTEMI, right bundle branch block, non-specific ST-T wave abnormalities, sinus tachycardia	Electrocardiogram, Transthoracic Echocardiogram, 18F-FDG PET/CT, Coronary Artery Catherization	5/8 admitted to the hospital due to cardiorespiratory symptoms and diagnosed with SLE. The other 3 patients diagnosed with SLE but no cardiorespiratory symptoms (1 admitted to dyspnea upon later questioning).	High-dose steroids (1 mg/kg prednisone) for all patients. 7/8 treated with mycophenolate motefil, 1/8 with cycophosphamide for lupus nephritis. 1 patient only got 3 days of pulse solumedrol IV (1000 mg)	3/8 Chest Pain, 1/8 Chest Pain and Palpitations, 2/8 Shortness of Breath	18F-FDG PET/CT: diffuse myocardial uptake → myocarditis. 50% with decreased EF. Median SLEDAI-2K: 5 (2–18). Median SLICC SDI: 0.5 (0–5)	Antinuclear antibodies, anti-SSA/Ro, anti-SSB/La, anti-ds-DNA, anti-Sm, anti-RNP, antiphospholipid antibodies. C3 and C4 levels, ESR, high-sensitivity C-reactive protein, troponin and pro-beta-natriuretic peptide (pro-BNP) levels	Low	18F-FDG–PET/CT imaging may be an effective way to diagnose clinical and subclinical myocarditis in SLE patients.
du Toit et al. (2021) [26]	Prospective cohort study	49 in original cohort but follow-up assessment done for 36 (32 F)	29	421 days (median)	Myocarditis	2D Echocardiogram, Cardiac MRI	HTN (19.4%), Diabetes (2.8%), Antiphospholipid Syndrome (16.7%)	At baseline: Antihypertensive (27.8%), Warfarin (2.8%), Chloroquine (44.4%), Immunosuppressive Treatment (16.7%), Prednisone. At follow-up: Antihypertensive (44.4%), Warfarin (22.2%), Chloroquine (94.4%), Immunosuppressive Treatment (22.2%), Prednisone.	Not Mentioned	Baseline CMR: 9 injury + clinical lupus myocarditis. 14 injury w/o clinical lupus myocarditis. Significant improvement after intensified immunosuppression: Decrease in SLEDAI-2K (13 → 7, p<0.001). Increase in LVEF, RV function, wall motion, and CMR wall index. No improvement: strain analyses, LV internal diameter index, myocardial tissue injury parameters. Improvement in CMR mass index ↔ reduction in myocardial edema	Complete blood count, inflammatory markers, C3, C4, markers of cardiac myocyte injury (creatine kinase (CK); high-sensitive troponin T [hs-TropT]), renal function, urine analysis, autoantibody screen (ANA, anti-dsDNA, anti-Sm, anti-RNP, Anti-Ro/SSA, Anti-La/SSB, ACA)	Low	Presence of CMR evidence of myocardial injury did not correlate with disease activity, serological markers, or cardiac function. Routine CMR may not be a useful tool, as any detected subclinical myocarditis had no significant prognostic implications.
Gioti et al. (2019) [27]	Case report	1 F	44	Not Mentioned	Periaortitis, diffuse subendocardial vasculitis (DSV)	ECG, Echocardiogram, Coronary Angiogram, CT, Cardiac MRI	Diagnosis of SLE without major organ involvement (digital vasculitis, arthritis and compatible serology [positive antinuclear Abs, increased anti-ds DNA Abs and low levels of C3, C4]). SLE in long-standing quiescence. Obstetric APS.	Low-dose Aspirin, Azathioprine, Hydroxychloroquine, Prednisolone, Methylprednisolone, Cyclophosphamide, Carvedilol, Ramipril	Patient presented to the ER with low back and abdominal pain lasting 3 days, along with an episode of vomiting. She also had dizziness and mild chest upon the second day of hospitalization.	Echo: diffuse hypokinesis (inferior, lateral, posterior walls), LVEF 40% CT: pneumonitis. Good response to immunosuppressive therapy	serum aspartate aminotransferase (AST), creatine phosphokinase (CPK), creatine kinase-MB (CK-MB), troponin, aPl, anti-dsDNA, C4, IgG4, ANCAs, High ESR, and CRP	Low	Periaortitis and DSV are rare but possible complications of a lupus flare that should be included in the differential. CMR was crucial to the diagnosis of DSV.
Wan et al. (2022) [28]	Cross-sectional observational	151 (86% F)	35.10 - control group, 37.33 - SLEDAI ≤ 4 group (inactive disease), 33.06 - SLEDAI ≥ 5 group (active disease)	81.50 months in SLEDAI ≤ 4 group, 72.89 months in the SLEDAI ≥ 5 group	Postsystolic shortening (PSS) and early systolic lengthening (ESL)	Two-dimensional speckle-tracking Echocardiography (2D-STE)	SLEDAI < 4: diabetes (2.56%), HTN (7.69%). SLEDAI ≥ 5: diabetes (4.88%), HTN (34.15%)	Glucocorticoids were the most common treatment (84.62% in SLEDAI < 4 and 97.56% in SLEDAI ≥ 5)	Not Mentioned	Peak systolic strain (PSImax), end-systolic strain (ESImax), EDV, end-diastolic volume index (EDVI), ESV, and end-systolic volume index (ESVI), and left ventricular mass index (LVMI) were significantly higher in patients with SLEDAI ≥5 compared to those with SLEDAI <4 and healthy controls. Global longitudinal strain (GLS) did not differ between SLEDAI ≥5 and ≤4 groups but was significantly lower in SLEDAI ≥5 versus healthy controls.	Not Mentioned	Low	2D-STE measurements of PSS and ESL may be useful markers for identifying subclinical myocardial dysfunction in SLE patients with preserved LVEF. The absolute value of GLS may not be a useful measurement.
Du Toit et al. (2021) [29]	Cross-sectional observational	41 (87.8% F)	29	Median of 158 days, median SLEDAI-2K of 13	Myocardial injury	2D-echo, CMR	Haematological (75%), Mucocutaneous (65.9%), Musculoskeletal (46.3%)	Chloroquine, Prednisone, Cyclophosphamide (n = 4), Mycophenolate Mofetil (n = 2), Azathioprine (n = 5), Methotrexate (n = 4), Sulfasalazine (n = 1)	Not Mentioned	46.3% (n=19) of patients met ≥1 CMR criteria for myocardial injury. Patients with CMR evidence of myocardial injury had significantly higher IL-18, IL-1Ra and IL-17 than those without. IL-1Ra predicted inflammatory and necrotic/fibrotic stages of myocardial injury on CMR.	inflammatory markers (CRP, ESR), C3, C4, markers of cardiac myocyte injury (creatine kinase (CK); high-sensitive troponin T (hs-tropT)), and urine analysis. An autoantibody screen included ANA, anti-ds DNA, anti-Sm; anti-RNP, anti-Ro/SSA, anti-La/SSB and antiphospholipid antibodies. SSA/Ro60 antigens. Cytokine levels (IL-1β, IL-1Ra, IL-2, IL-6, IL-10, IL-17, IL-18, TNF-alpha), markers of endothelial activation (sVCAM-1), and myocyte strain (sST2)	Low	Serum IL-18 and IL-1Ra may be useful biomarkers for identifying subclinical myocardial injury.
Yerram et al. (2023) [30]	Cross-sectional observational	75 F, 15 M	27.6	27 months, mean SLEDAI of 19.3	myocarditis, other cardiovascular complications not specified	Not Mentioned	Not Mentioned	Not Mentioned	Hospitalised for Acute Severe Lupus	SLEDAI, anti-dsDNA titres, and serum IFN-α levels were significantly higher in non-survivors. C3 levels were significantly lower in non-survivors. Serum IFN-alpha levels better predicted mortality than these other markers.	Anti-dsDNA, C3, C4, INF-alpha	Low	Highlights the role of INF-alpha as a potentially important biomarker to predict in-hospital mortality in a lupus flare
Saleem et al. (2022) [31]	Case report	1 F	60	SLEDAI of 8	Pulmonary Arterial HTN (PAH)	Electrocardiogram, Computed tomography Angiography with contrast, Duplex Ultrasound venous lower extremity, Transthoracic Echocardiography	Unprovoked Pulmonary Embolism (2 years ago)	Apixaban, Furosemide, Hydroxychloroquine, Steroids, Sildenafil	Exertional Dyspnea, Generalized Weakness, Lower Extremity Swelling, Orthopnea, Occasional Paroxysmal Nocturnal Dyspnea, Pleuritis, Lymphopenia	Not Mentioned	ANA, high B-type natriuretic peptide, D-dimer, ESR, CRP, anti-ds DNA Ab	Low	Standard SLE-aPAH therapy: immunosuppressants (cyclophosphamide, rituximab, steroids) and prostanoids. For patients with an intrapulmonary shunt and minimal disease activity, hydroxychloroquine and sildenafil are an effective alternative.
Yilmazer et al. (2015) [32]	Case report	1F	24	7 years	Profound sinus bradycardia	Classical 12-lead ECG and Holter ECG	Sinus node dysfunction; Lupus nephritis	High-dose Methylprednisolone, Cyclophosphamide (1000 mg), Hydroxychloroquine 200 mg/d, and Prednisolone 5mg/d	Fever, Generalized Myalgia, Arthralgia, Photosensitivity, Facial Rash, Arthritis, Dizziness	Not Mentioned	anti-dsDNA Ab (initially positive and later was negative); white blood cell count, 10800/mm 3; C-reactive protein, 2.1 mg/dL [0 – 0.5]; C3, 148 [90 – 180 mg/dL], hypocomplementemia, 24-h urine protein, 1397 mg/day	Low	Isolated profound bradycardia is a rare but possible complication of a lupus flare. Clinicians should be vigilant of conduction systems abnormalties due to lupus flare and take appropriate measures, including a full cardiovascular exam and eCG monitoring.
Raval et al. (2021) [33]	Case report	1 M	20	6 months	Bilateral pleural and pericardial effusions with dense consolidations, LVEF < 10%, diffuse myofiber degeneration and inflammation	Electrocardiogram, CT chest, Transthoracic and Transesophageal Echocardiogram, Cardiac MRI, Endomyocardial Biopsy	MSSA bacteremia	Hydroxychloroquine, Broad-spectrum IV Antibiotics, and Corticosteroids	Acute Dyspnea, Pleuritic Chest Pain, Fever, Generalized Fatigue, Dry Cough, Chronic Diffuse Progressive Rash	Not Mentioned	hypocomplementemia, positive ANA with anti-Sm, anti-RNP, chromatin, SS-A, dsDNA, perinuclear anti-neutrophil cytoplasmic antibodies (pANCA), myeloperoxidase, and troponin T	Low	SLE myocarditis is a known but rarely fatal cause of cardiogenic shock. Prompt diagnosis via serology, imaging, and biopsy is critical, as shown by a case of de novo cardiogenic shock that rapidly improved.
Giollo et al. (2022) [34]	Case Control	59 (27 patients (89% F) and 32 controls (100% F))	45 ± 11 (patients). 46 ± 7 (controls)	14 years; 26% patients having a SLEDAI-2K of zero	Antiphospholipid antibody syndrome (19%), myocardial fibrosis (19% patients, 0% controls)	Echocardiography with Ultrasound multi-pulse scheme (eSCAR) and speckle tracking Echocardiography	HTN (30% patients, 9% controls); Hypercholesterolemia (15% patients, 19% controls)	Hydroxychloroquine, Immunosuppressants (Methotrexate, Azathioprine, Mycophenolate, Belimumab), and Glucocorticoids	Arthritis (74%), Mucocutaneous manifestations (59%), Lupus Nephritis (44%)	Not Mentioned	decreased serum C3 or C4 (56%), and anti-dsDNA antibodies (74%)	Low	The eSCAR technique efficiently detects subclinical heart damage in SLE patients, predicting flares, and is suitable for routine cardiac surveillance due to being easy, cheap, fast, and well-tolerated.
Gusetu et al. (2016) [35]	Case Control	148 (75 patients (89.3% F) and 73 controls (91.8% F))	43.2+/-12.5 patients. 41.8+/-11 controls	8.03 (6.3) years	SLE patients had a significant decrement in endocardial longitudinal strain (-18.4% vs 19.3%); Diastolic dysfunction (DD) (45.3%), Antiphospholipid Syndrome (APS) (28%), Pericardial effusion (25.3%), Endocarditis (4.0%), Pleural effusion (24.0%)	Transthoracic Echocardiography (tissue Doppler imaging and speckle tracking Echocardiography)	Hypertension (26.6%), Diabetes (5.3%), Dyslipidemia (45.3%), Obesity (22.6%), Renal (34.6%), Neuropsychiatric (22.6%), Skin (58.6%), Raynaud's phenomenon (13.3%), Musculoskeletal (85.3%), Eye (4.0%), Vasculitis (14.6%), Sjogren's syndrome (10.7%)	Follow up with Maternal-Fetal Medicine	Not Mentioned	APS with DD (55.9%) APS without DD (4.9%). APS induces myocardial hypoperfusion via microvascular thrombosis and small vessel vasculopathy, ultimately impairing myocardial compliance (diastolic dysfunction)	Anti-Ro antibodies in 37% of the SLE pts	Low	Early cardiac impairment in SLE is common without CVD and driven by organ damage, disease duration, APS, and hypertension
Santos et al. (2023) [36]	Case Report	1 F	27	2 years	Paroxysmal Atrial Fibrillation, Myocarditis	Electrocardiogram, Computed Tomography Angiography, Transthoracic Echocardiogram,	Lupus	Mycophenolate, Hydroxychloroquine, Metoprolol, Mycophenolate, Prednisone, Steroid Taper, Solumedrol	bilateral focal neurological deficits in the arms and legs, hemodynamically stable and afebrile, extremity weakness,	Myocarditis diagnosis relies on clinical suspicion, especially in subclinical cases, based on high-sensitivity troponins, lupus flare labs, and episodes of atrial fibrillation.	CSF protein, CSF glucose, RBCs, lymphocytes, monocytes, creatinine kinase, ANA, Sjogren's antibody, DNA DS antibody, atypical p-ANCA antibody, SS-A, SS-B, Ab IgG, RNP Ab IgG, Scl 70 Ab IgG S, Jo 1 Ab IgG S, C3, C4, Ds-DNA Ab, Cardiolipin antibody IgA, Cardiolipin Antibody IgM, Cardiolipin antibody IgG, Troponin	Low	Active lupus can be a differential diagnosis for atypical presentations of cardiac and neurological involvement, so recognizing atypical manifestations is important for routine vital care. Instruments such as PGA and SELENA-SLEDAI can help assess worsening lupus activity.
Zinglersen et al. (2025) [37]	Longitudinal Cohort Study	147 (88.7% F)	47	14 years	Coronary Artery Calcification, Myocardial Infarction, One required coronary artery bypass graft	CT imaging of the coronary arteries, Invasive Coronary Angiography	Myocardial Infarction (7 patients), Lupus Nephritis (58%), HTN, Hypercholesterolemia	Not Mentioned	Not mentioned	CAC progression was not clearly linked to MI, possibly due to a few events. Some MI in SLE may be atherosclerotic or SLE-related thrombosis. Two SLE patients with MI but no CAC were positive for lupus anticoagulant (LAC).	Coronary Artery Calcification volume score, SDI score, SLEDAI-score, eGFR, aPL, LAC	Low	The study showed that progression of coronary artery calcification is associated with smoking, duration of SLE disease, and previous presence of CAC, but was inconclusive for associations between renal invovlement and incidence of MI.
Tornvall et al. (2021) [38]	Retrospective Cohort Study	4198 (83.2% F)	55 +/- 18	Not Mentioned	Acute Myocardial Infarction, Myocardial Infarction with Nonocclusive coronary arteries	Coronary Angiography	Hypertension (9.8%), Diabetes Mellitus (5.2%), Renal Disease (1.3%), Hyperlipidemia (0.3%), Obesity (0.5%), COPD (1.6%), Liver Cirrhosis (0.7%), Mental Disorders (6.6%)	Not Mentioned	Not mentioned	The causes for the higher incidence in this study could be from an older SLE cohort and a longer follow-up. Another difference that may have affected the results is that Canadian and Taiwanese studies followed incident cases, whereas prevalent cases were studied before 1996.	Not Mentioned	Low	In this patients of SLE, there is increased incidence of acute myocardial infarction in patients with Lupus, but there is no direct indication that the proportion of myocardial infarction in non-occlusive coronary arteries is increased in SLE. It is important to investigate patients with SLE with acute myocardial infarction with coronary angiography.
Nikdoust et al. (2018) [39]	Cross Sectional	33 F	27	2 years	Not Mentioned	Global Longitudinal Strain, Speckle Tracking Echocardiography	Lupus	Not Mentioned	Not mentioned	Apical 2- and 3-chamber views showed significantly lower left ventricular global longitudinal strain (LV GLS) in SLE patients compared to healthy controls.	Left Ventricular Global Longitudinal Strain, Left Ventricle Ejection Fraction	Low	Global Longitudinal Strain within 2D speckle tracking echocardiography is a cost-effective, noninvasive method to check for cardiac complications in SLE, including lupus myocarditis. This could be considered in the routine care of atients for lupus to be cost-effective and beneficial to patients.
Yousif et al. (2022) [40]	Case Report	1 F	21	Not diagnosed before	Cardiac Tamponade, Tachycardia, Pericardial Effusion, Atrial Fibrillation, Pleuritic Chest Pain	Echocardiogram, CT pulmonary embolism protocol, Transthoracic Echocardiography, Chest X-Ray	Preeclampsia, HTN, Lupus Nephritis, Pregnancy	Prednisone, Solumedrol, Digoxin, Hydroxychloroquine, Trimethoprim/Sulfamethoxazole	28 weeks of gestation, severe chest pain for 10 days, left shoulder pain, chest pain was worse when lying flat with inspiration and better with sitting forward	Pericardial fluid: glucose 67 mg/dL, LDH 166 U/L, protein 4.7 g/dL → exudative effusion; urine protein: 100 mg/dL initially, >500 mg/dL next day	ANA, anti-Sm, anti-RNP, anti-chromatin, anti-SSA, anti-SSB, anti-dsDNA, and low C3, Lupus Anticoagulant, Anti-Cardiolpin antibody, ESR, BNP, complete metabolic panel	Low	If a patient has SLE and is not treated if they have symptoms of cardiac tamponade, this can create maternal and fetal complications, so monitoring and recognition are very important. In this case of SLE, the case started with pericardial effusion to lead to tamponade, and the patient had to be addressed so that there would be good maternal and fetal outcomes.
Ashour et al. (2023) [41]	Case Report	1 F	32	Not Mentioned	Sinus Tachycardia	Cardiac MRI, ECG, CT Pulmonary Angiogram	Viral Myocarditis 4 months prior	Bisoprolol, Piperacillin/Tazobactam, Lorazepam	progressive heart palpitations, generalized skin rash for 3 days, heart rate has reached 100-150 bpms	Echo: LVEF 54%, no pericardial effusion; nonspecific ECG	Negative Troponin, C-reactive protein, procalcitonin, and lactic acid, ANA, positive anti-dsdna, low C3 and C4	Low	Sinus Tachycardia is one of the common cardiac manifestations of SLE, but physicians should consider SLE flare in patients with sinus tachycardia without any other clear explanations.

Cardiac Manifestations in SLE

Pericardial involvement was frequently described, ranging from isolated pericarditis to pericardial effusion and, in severe cases, tamponade. For instance, a 35-year-old female with lupus nephritis developed cardiac tamponade requiring urgent intervention [11], while a large Chinese cohort reported pericarditis in 9.5% of patients [15]. Myocardial disease was also observed, with presentations of myopericarditis and myocarditis; one case of lupus myopericarditis in a young male mimicked acute coronary syndrome (ACS) [12], while larger studies identified myocarditis in approximately 5-6% of patients [15]. Left ventricular dysfunction was variably reported, with some cross-sectional cohorts identifying reduced systolic function on echocardiography. Valvular abnormalities, primarily regurgitant lesions, were observed in both retrospective and cross-sectional cohorts, with prevalence rates reported as high as 32% [13,15]. Arrhythmias and conduction defects were described in isolated cases, while pulmonary hypertension emerged as a relatively frequent complication, affecting up to 42% of patients in one cross-sectional study [13]. Due to heterogeneous reporting across studies, morbidity percentages for individual cardiac complications could not be uniformly extracted. While pulmonary hypertension was frequently associated with increased mortality, current evidence is insufficient to conclude whether it is the leading cause of death in SLE-related cardiac disease. However, we were able to extract the frequency of cardiac complications from case reports and case series (summarized in Table 2). Pericardial disease, myocardial disease, and arrhythmia were the most frequently reported complications. There was heterogeneity in myocarditis definitions, but most studies employed an imaging-based definition. Many patients present multiple complications. It should be noted that the only case series included in this review used 18-fluorodeoxyglucose positron emission tomography to identify myocarditis in five of its patients, thereby heavily influencing the percentage we calculated.

**Table 2:** Percentage of SLE patients with cardiac complications in case reports/series Source: Refs [11,12,16,20,21,24,25,27,31-33,36,40,41] SLE: Systemic lupus erythematosus

Complication	N (%)
Pericardial Disease	9 (50)
Myocardial Disease	9 (50)
Arrhythmia	9 (50)
Heart Failure/LV Dysfunction/Wall Abnormalities	6 (33.33)
Coronary Artery Disease	3 (16.66666667)
Valvular Disease	2 (11.11)
Vasculitis	2 (11.11)
Pulmonary Hypertension	1 (5.56)

The estimated prevalence of cardiac involvement varied substantially depending on study design and case ascertainment. Case reports and case series predominantly highlighted clinically overt events, including myocarditis and cardiac tamponade, whereas observational cohort studies more commonly identified subclinical abnormalities detected through echocardiographic screening rather than individual clinical symptoms. The percentages derived from case reports and case series (Table 2), therefore, reflect the wide spectrum of cardiac complications described in published cases.

Clinical Presentation

Patients frequently presented with chest pain, dyspnea, and fatigue [19]. Acute presentations, including pleuritic chest pain with diffuse ST-segment elevations and tamponade, were highlighted in case reports [40], while subclinical disease predominated in larger cohorts [15]. Importantly, case reports emphasized the overlap with ACS, as lupus cardiac involvement often presented with chest pain, dyspnea, and/or elevated cardiac biomarkers, creating diagnostic challenges [16,21]. The distribution of clinical presentations and symptoms reported in case reports and case series is summarized in Table 3, including both presenting symptoms and those that developed during hospitalization. All case reports described symptoms temporally associated with lupus flares, with the exception of one case series that included two subclinical cases, which are not included in the table.

**Table 3:** Distribution of clinical presentations in reported SLE sase studies/series The “cases” column represents the number of patients in case reports/series that presented with these symptoms (either self-report or found on physical exam) or developed them during their hospital stay. Source: Refs. [11,12,16,20,21,24,25,27,31-33,36,40,41]

Clinical Presentations Category	Symptoms	Cases
Cardiopulmonary Symptoms	Chest Pain	11
	Pleuritis	3
	Dyspnea	5
	Orthopnea	2
	Occasional Paroxysmal Nocturnal Dyspnea	1
	Cough	2
	Hemoptysis	1
	Bibasilar Rales	1
	Systolic Murmur	1
	Tachycardia	2
	Progressive Heart Palpitations	1
	Palpitations	1
Constitutional/General Symptoms	Fever	4
	Generalized Weakness	1
	Generalized Fatigue	1
	Generalized Myalgia	1
	Dizziness	2
	Vomiting	2
	Diarrhea	1
Musculoskeletal Symptoms	Arthralgia	2
	Lower Leg Pain	1
	Arthritis	1
	Bilateral Shoulder and Hip Tenderness	1
	Low Back and Abdominal Pain	1
Neurological Symptoms	Seizures	1
	Bilateral Focal Neurological Deficits in the Arms and Legs	1
	Extremity Weakness	1
Dermatologic Symptoms	Rash	3
	Photosensitivity	1
Hematologic/Immune Findings	Lymphopenia	1
Swelling/Edema	Limb Swelling	2
	Left Shoulder Pain	1
	Polyarticular Effusions	1

A number of case studies noted delayed recognition of SLE-related cardiac events, particularly in patients presenting with neurological/constitutional symptoms, arrhythmias without chest pain, or gradually developing symptoms [27,36,41]. Several studies and case reports described patients with both lupus nephritis and cardiac complications; however, due to heterogeneity in reporting and lack of subgroup analysis, the exact prevalence could not be determined [11,17,20,25,32,34,37]. Cardiac involvement was also noted in patients presenting with other lupus flares, but data directly correlating the severity of nephritis with cardiac outcomes were limited. Additionally, no clear relationship was identified between coronary artery calcification and lupus disease stage. This gap highlights an important area for future research, particularly to explore whether the severity of lupus nephritis correlates with the extent or progression of cardiac complications in SLE.

Diagnostic Findings

Echocardiography was the most commonly employed diagnostic modality, especially conventional transthoracic echocardiography (2D and Doppler imaging). Conventional echocardiography was primarily assessed for pericardial effusion, pulmonary artery pressures, valvular abnormalities, and left ventricular function.

Conventional echocardiographic findings, including pericardial effusion, ranged from 13.6% (8/59) to 25.3% (19/75) across cohorts [14,35]. Diastolic dysfunction was observed in up to 45.3% (34/75) [35], while systolic dysfunction and reduced left ventricular ejection fraction (LVEF) were also reported in multiple studies [14,18,19,21,26,27,33,41], with some noting LVEF <50% in 38% (30/79) in lupus myocarditis patients and LVEF <10% in severe cases [18,33]. Cross-sectional studies typically capture ventricular dysfunction at a singular timepoint (during flare-related admission) without data on changes in LVEF or diastolic dysfunction during follow-up. However, one case cohort study noted a significant improvement in echocardiographic LVEF at 12-month follow-up (p=0.014) [26], and most case reports described great improvement in echocardiographic LVEF on follow-up visits [27,33,41]. Endocarditis was infrequently reported, with one study noting a low prevalence of 0.15-4% [23]. Other conventional echo-related findings included abnormal ventricular wall motion and LA dilation, though data on these were limited. These findings highlight the heterogeneity of cardiac involvement in SLE and suggest a need for more standardized echocardiographic assessment in future studies.

Multiple studies used advanced echocardiography. Most commonly, they used 2D-speckle tracking echocardiography to identify markers of subclinical cardiac disease, such as abnormalities in global longitudinal strain, post-systolic shortening, and end-systolic lengthening [22,32-34,38]. Giolla et al. used scar-imaging echocardiography with an ultrasound multi-pulse scheme (eSCAR) to identify myocardial scars in SLE patients without cardiac symptoms or history of cardiac disease [34]. They found that e-SCAR patients had a significantly higher probability of having a lupus flare over a one-month follow-up (p=0.0001), highlighting a potential role for echocardiographic methods to identify early cardiac warning signs and prevent serious complications.

Although less common than echocardiography alone, some case reports also described using cardiac MRI (CMR), chest CT, or coronary CT angiography in addition to echocardiography. CMR was used to evaluate for potential lupus myocarditis in both case reports and cohort studies [27,29,41] or confirm echocardiographic findings [24]. In one case report, CMR was crucial to diagnose diffuse subendocardial vasculitis over lupus myocarditis [27] and helped find a thrombus that was not present on other imaging [21]. Coronary CT angiography and chest CT were found to be helpful to rule out ischemic etiologies, namely, acute coronary syndrome or pulmonary embolism [24,27,31,33].

Laboratory markers, such as troponin, ESR, CRP, and complement levels, were frequently measured, with active disease correlating with elevated inflammatory markers and reduced complement. Autoantibody profiles, including anti-dsDNA, anti-RNP, and anti-SSA, were variably reported across studies, ranging from elevated to normal levels. A couple of studies focused on studying more novel biomarkers. du Tuit et al. found that serum IL-18 and IL-1Ra were significantly elevated in SLE patients with myocardial injury, and IL-1Ra independently predicted stages of CMR myocardial injury [28]. In Yerram et al.'s study, serum IL-1 alpha levels predicted in-hospital mortality better than SLEDAI, anti-dsDNA, or complement levels [23].

Risk Factors and Comorbidities

Several comorbid conditions included lupus nephritis, hypertension, and dyslipidemia [21,35], with one cross-sectional study noting that over half of participants had at least one atherosclerotic risk factor [17]. Thyroid disease and anemia also appeared as incidental findings in case reports and cross-sectional studies. Associations with comorbidities mostly came from descriptive reporting, as opposed to multivariable adjusted analyses. High SLEDAI scores were associated with cardiac manifestations, and severe disease activity emerged as a recurring risk factor [18,30]. Disease duration was variably reported in larger analyses, and most studies did not look at whether disease duration independently contributed to cardiac risk beyond disease activity. Younger age and female sex predominated in most studies, consistent with the epidemiology of SLE, though severe manifestations such as myopericarditis were also observed in males [12,17].

While corticosteroid use was common across studies, with up to 95% of patients receiving them [29], there was limited direct data correlating steroid use or dose with increased cardiovascular complications. However, some studies suggested that high-dose steroids (e.g., ≥1 mg/kg prednisone or IV methylprednisolone pulses) were used to manage severe cardiac manifestations, such as lupus myocarditis [24,25], while others advised early steroid-sparing strategies due to the potential role of steroids in accelerating atherosclerosis [13]. Although glucocorticoids are central to the acute treatment of SLE flares, chronic use is associated with increased cardiovascular risk, emphasizing the importance of balancing immediate disease control against long-term cardiovascular outcomes. Other risk factors associated with cardiovascular complications included hypertension, smoking, dyslipidemia, obesity, and longer disease duration. Socioeconomic factors, such as food insecurity or income, were not consistently reported.

Management Strategies

Immunosuppressive therapy, particularly corticosteroids, was the cornerstone of treatment, often in combination with agents such as hydroxychloroquine, mycophenolate mofetil, or cyclophosphamide. Biologic therapies such as belimumab, rituximab, and voclosporin were reported less frequently. Cardiac-specific interventions included beta-blockers, angiotensin-converting enzyme (ACE) inhibitors, and colchicine for pericardial involvement. Procedural interventions were rarely required but included pericardiocentesis in cases of tamponade [11,16,40]. Surgical approaches were not commonly reported. Table 4 summarizes the number of patients who received each medication in case studies/series.

**Table 4:** Distribution of medications by class and frequency in case studies/series TMP/SMX: trimethoprim/sulfamethoxazole Source: Refs [11,12,16,20,21,24,25,27,31-33,36,40,41]

Drug Category	Medication	Number of Patients
Glucocorticoids (Corticosteroids)	Hydroxychloroquine	16
	Prednisone	9
	Steroids	5
	Methylprednisolone	3
	Prednisolone	2
Immunosuppressants and DMARDs	Mycophenolate	4
	Cyclophosphamide	4
	Azathioprine	2
	Rituximab	1
	Belimumab	1
	Voclosporin	1
Anticoagulants	Carvedilol	4
	Low-dose aspirin	3
	Metoprolol	2
	Warfarin	1
Beta Blockers	Bisoprolol	1
	Apixaban	1
	Atenolol	1
Calcium Channel Blockers	Nifedipine	1
ACEi and ARBs	Lisinopril	1
	Ramipril	1
	Valsartan	1
Antihypertensives and Vasodilators	Hydralazine	1
	Isosorbide Mononitrate	1
Antibiotics	Piperacillin/Tazobactam	1
	TMP/SMX	1
Analgesics and Pain Medications	Oxycodone	1
Others	Atorvastatin	3
	Digoxin	1
	Lorazepam	1
	Sildenafil	1
	Methimazole	1

Outcomes and Prognosis

Outcomes varied according to the severity and type of cardiac involvement. Case reports demonstrated resolution of symptoms with appropriate immunosuppressive and supportive therapy, although a case report of a 35-year-old female reported recurrence of pericardial effusion [11]. In larger cohorts, pericardial effusion and pericarditis were generally responsive to therapy [14], while pulmonary hypertension and myocarditis were associated with worse outcomes [13,15]. Mortality was not systematically reported but was linked to severe complications and high disease activity. Predictors of adverse outcomes included lupus nephritis, elevated disease activity indices, and coexisting cardiovascular risk factors [17,34,37].

Key Takeaways

Cardiac involvement in SLE is heterogeneous, ranging from mild pericarditis to life-threatening tamponade, myocarditis, and pulmonary hypertension. Subclinical disease was common, underscoring the importance of routine screening with echocardiography and biomarkers. Early recognition and prompt initiation of immunosuppressive therapy remain critical to improving outcomes. For clinicians, these findings emphasize the need for vigilance in evaluating cardiac symptoms in SLE patients and for integrating cardiovascular monitoring into long-term management strategies.

Discussion

This review highlights the broad spectrum of cardiac involvement in SLE, ranging from mild pericardial disease to severe and potentially fatal complications, such as tamponade, myocarditis, and pulmonary hypertension. Across diverse geographic settings and study designs, cardiac manifestations were consistently reported, reinforcing that cardiovascular involvement remains a major contributor to morbidity in SLE. Importantly, many cases were subclinical and only detected through specialized imaging, suggesting that the true prevalence of cardiac disease in SLE may be underestimated.

In the included studies, two main patterns of SLE-related heart disease emerged, with some overlaps. The first includes inflammatory cardiac manifestations, including pericarditis, myocarditis, myopericarditis, and vasculitis, resulting in injuries. The second includes ischemic heart disease manifestations, such as myocardial infarction and acute coronary syndrome. These patterns are difficult to separate because inflammatory conditions such as lupus myocarditis or myopericarditis can present with common acute coronary symptoms, such as chest pain and elevated cardiac biomarkers. Therefore, advanced imaging such as cardiac MRI is frequently required to accurately and carefully interpret the results.

Underrecognition of cardiac involvement in SLE appears to stem from two key issues. First, many patients have subclinical disease that is not evident through symptoms and is only detected on imaging, such as echocardiography. Second, as reflected in case reports and case series, cardiac complications are often recognized only after the disease has progressed or acute symptoms develop, likely reflecting the absence of standardized screening protocols.

Although several cardiac manifestations were primarily detected via specialized imaging, others, including myocarditis and pulmonary hypertension, were associated with poorer prognosis and higher risk of death. Some subclinical imaging findings, such as eSCAR positivity, correlated with short-term disease activity, including an increased likelihood of lupus flares. These observations underscore the need for longitudinal studies to determine whether subclinical imaging abnormalities predict long-term outcomes such as heart failure or mortality.

Substantial gaps exist in cardiovascular risk stratification for patients with SLE. No consensus framework currently defines low-, moderate-, or high-risk patients. Commonly used tools, such as coronary artery calcium scoring, may miss inflammatory plaque features, emphasizing the need for SLE-specific cardiovascular risk assessment strategies that integrate advanced imaging modalities.

Our findings align with prior literature indicating that pericardial disease is the most common cardiac manifestation of SLE, with effusion and pericarditis reported in up to 50% of patients in some cohorts [13,42]. This prevalence highlights the diagnostic challenge for clinicians, as distinguishing lupus myocarditis or myopericarditis from ischemic heart disease is critical but often difficult without advanced imaging.

Pulmonary hypertension emerged as another important complication, with some cross-sectional studies reporting prevalence as high as 40% [13]. Given its association with increased mortality, this underscores the clinical importance of early detection of valvular disease and arrhythmias, which, while less frequently documented, were observed and warrant further attention in future studies [13,15].

Identified risk factors and comorbidities, including lupus nephritis, hypertension, and elevated disease activity scores, support the role of systemic inflammation and immune dysregulation in cardiac injury [34]. The predominance of female patients reflects the epidemiology of SLE; however, severe cardiac disease in males suggests that sex alone should not dictate screening practices.

Management strategies centered on immunosuppressive therapy, particularly corticosteroids, with adjunctive agents, such as hydroxychloroquine, mycophenolate, and cyclophosphamide, were employed selectively [14]. Colchicine was occasionally applied in pericardial disease, reflecting adaptations from non-SLE populations. Procedural interventions were rare but lifesaving in cases of tamponade [12]. Despite these strategies, outcomes for patients with myocarditis and pulmonary hypertension remain guarded, underscoring an urgent need for more effective therapeutic approaches.

Taken together, these findings highlight several important clinical considerations. Targeted cardiovascular evaluation, including echocardiography, ECG, and biomarkers such as troponin, CRP, ESR, complement levels, and autoantibodies (e.g., ANA, anti-dsDNA), may be useful for SLE patients at higher risk, though the optimal timing, frequency, and selection criteria remain unclear. Additional tools, such as lipid profiles, lipoprotein(a), high-sensitivity CRP, and coronary CT calcium scoring, could further refine risk assessment, but their routine use in all SLE patients is not yet supported by evidence. Clinicians should also be aware of the overlap between lupus myocarditis and ischemic syndromes to prevent diagnostic delays. Finally, longitudinal studies are urgently needed to define predictors of adverse outcomes, evaluate the durability of treatment responses, and clarify the role of novel biologic therapies in preventing or mitigating cardiac involvement in SLE.

Limitations

This review is limited by the heterogeneity of the included studies, which varied in design, sample size, and reporting standards. Case reports and small cohorts provided detailed clinical descriptions but limited generalizability, while larger retrospective studies often lacked detailed biomarker or imaging data. Additionally, the available literature is subject to publication bias, as cases with more dramatic or unusual outcomes are more likely to be reported. There may be underrepresentation of cases from low-resource settings where diagnostic and reporting capabilities might be limited. Studies differed significantly in the imaging modalities employed, with some using nuclear medicine or cardiac MRI and others limited to 2D-echo, which affected the sensitivity for detecting cardiac conditions. Any recommendations for routine screening should be interpreted with caution, as the available evidence from the included studies is limited. Furthermore, geographic variability may influence the observed prevalence of cardiac complications.

Future Directions

Next steps should focus on creating clear, consensus-based guidelines in three main areas. First, defining a streamlined approach for initiating and continuing cardiovascular monitoring in patients with SLE, including routine ECGs and echocardiography, and determining when to incorporate advanced imaging if initial findings are normal. Second, integrating biomarker testing into standard care to identify cardiovascular risk earlier, these could include troponin, CRP/ESR, autoantibodies, and other emerging markers. Third, developing a validated, SLE-specific risk assessment for atherosclerosis, which could improve risk stratification, help identify subclinical disease, and better link ischemic heart disease and myocardial involvement in this population. Prospective studies, registries, and validation of risk scores in diverse cohorts will be essential to translate these approaches into evidence-based clinical practice. Future research should include prospective studies to define the benefits, optimal timing, and target populations for systematic screening. Development of validated risk scores could help identify patients most likely to benefit from early intervention. Cohort studies or registries are also needed to clarify the long-term impact of advanced imaging and biomarkers on clinical outcomes.

## Conclusions

Cardiac manifestations are a frequent and clinically significant complication of systemic lupus erythematosus, with pericarditis, myocarditis, and pulmonary hypertension representing the most important contributors to morbidity. Many cases remain subclinical and undetected without routine imaging, yet others present with acute, life-threatening complications that mimic ischemic disease. Early recognition and aggressive treatment with immunosuppressive therapy are critical for improving outcomes. Moving forward, systematic screening, multidisciplinary collaboration between rheumatology and cardiology, and the integration of advanced imaging and biomarker strategies will be essential to reduce the burden of cardiac disease in SLE.
